# Accuracy of intracranial pressure assessment with a non-invasive transcranial doppler and arterial blood pressure method in patients with suspected idiopathic intracranial hypertension

**DOI:** 10.1186/s13089-025-00434-4

**Published:** 2025-06-27

**Authors:** Nabil Al Shammas, Sophie Schumann, Dragana Köhler, Bernhard Rosengarten

**Affiliations:** Department of Neurology, Chemnitz Medical Center, Dresdner Str. 178, 09131 Chemnitz, Germany

**Keywords:** Non-invasive intracranial pressure, Optic nerve sheath diameter, Empty sella, Pseudotumor cerebri, Idiopathic intracranial hypertension, Lumbar puncture, Transcranial doppler ultrasonography

## Abstract

**Objective:**

The incidence of idiopathic intracranial hypertension (IIH) has nearly doubled in the recent decade, possibly due to increasing obesity rates. Lumbar puncture pressure (LPP) assessment is still the diagnostic gold standard but due to invasiveness of the method, several non-invasive alternatives exist. We evaluated a non-invasive intracranial pressure (nICP) method for its accuracy to predict LPP.

**Methods:**

Prospectively, we included patients with suspected IIH and obtained nICP by means of a combined bilateral transcranial Doppler and photoplethysmographic arterial blood pressure method. In addition, we searched for an empty sella sign by magnetic resonance tomography and evaluated the optical nerve sheath diameter (ONSD) bilaterally by Duplex sonography. We analyzed data on an individual level for their capability to predict LPP. Included were 70 patients from which 60 with a complete data set were used for further evaluation. Patients with symptomatic intracranial pressure were excluded.

**Results:**

The nICP and LPP correlated with *R* = 0.85 on the right, and *R* = 0.79 on the left side (*p* < 0.001, respectively). The mean difference of nICP-LPP was 0.45 ± 4.93 cmH_2_O. Its sensitivity to predict an increased ICP was 0.92, the specificity was 0.88 and negative predictive value 0.88. The empty sella sign and the ONSD showed no significant correlation to the LPP.

**Conclusion:**

The nICP method allows pre-diagnosis of increased ICP and might help in decision making for the need of LPP. Due to the moderately increased ICP levels, ONSD remained insignificant.

## Introduction

Idiopathic intracranial hypertension (IIH) usually occurs in obese women of childbearing age [1], although pediatric cases are not uncommon [2]. The typical symptoms are headache, sight impairment and vertigo. In case of persistently increased intracranial pressure (ICP), the risk of permanent visual loss is frequent [3]. From 2003 to 2017 the incidence of IIH increased three times to 7.8/100,000/y, possibly corresponding to population increases in obesity [4]. Lumbar puncture pressure (LPP) assessment is still the gold standard for diagnosis, therapy, and follow-up investigations of IIH patients. Due to its invasive and sometimes painful nature, patients do not always tolerate lumbar puncture. Moreover, fear and stress may cause an unstable or false-positive assessment of lumbar pressure. Therefore, LP should be used at minimum, which is only for therapeutic reasons to withdraw liquor in case of increased ICP. Different techniques using different concepts are used to estimate ICP non-invasively: Duplex-sonography related measurement of the optic nerve sheath diameter (ONSD) [5, 6], appearance of an empty sella [7–9] or a stenosis of the sinus transversus in cerebral MRI scans [10] were used to give information on ICP. Although these and other, MRI-based, techniques [11, 12] are used in clinical practice, they do have some limitations regarding their accuracy [13, 14]. Recently, we validated an established technique from neurocritical care [15–19] to measure the ICP noninvasively (nICP) [20, 21]: nICP was calculated from simultaneous recording of the intracranial cerebral blood flow velocity (CBFV) with transcranial Doppler (TCD) ultrasound and arterial blood pressure (ABP) with a photoplethymographic technique.

In the present study we used this combined TCD-ABP method in patients with suspected IIH to assess diagnostic accuracy of the technique to predict the LPP. We also obtained the ONSD and the empty sella sign as additional parameters.

## Materials and methods

### Patients

In this prospective study, 70 consecutive patients that were treated in our hospital for suspected IIH were included in the study. All patients presented clinical symptoms of idiopathic intracranial hypertension according to the consensus recommendations of Mollan et al. [22]: chronic headaches, ophthalmologic changes and/or vertigo plus attention impairment, chronic fatigue. Patients underwent cerebral MRI, ophthalmoscopy, and LP. Body mass index (BMI) was calculated from each patient. ONSD and nICP were assessed one hour prior to LP. MRI was performed maximal 2 d before LP. If the LPP was above 20 cmH_2_O a lumbar drainage of 10–30 ml of CSF was performed. This was a part of our institutional protocol for management of IIH [23]. Excluded were patients when concurring findings other than IIH occurred in the diagnostic workup, such as stenosis of cerebral veins or sinus, or other diagnoses such as migraine or tension type headache. Excluded were also patients with stenosis/occlusion of extra/intracranial vessels or cardiac arrhythmia.

### Evaluation of the nICP

nICP was assessed in a supine position on a comfortable diagnostic chair. CBFV was assessed by transcranial Doppler (TCD) using a 2-MHz pulsed Doppler monitoring probe (Delica EMF-9 d pro, Shenzen Delica Medical Equipment Co., China). CBFV was obtained from both middle cerebral arteries (MCA) in a depth of about 55–65 mm. TCD probes were secured in place by using a headset provided by the device manufacturer. ABP was continuously and non-invasively measured with a photoplethysmographic cuff method (Finapres NOVA, Finapres Medical Systems BV, Enschede, The Netherlands), placed around a finger. The measuring level of the ABP was adjusted to the level of the MCA. Simultaneously assessed TCD and ABP data were further calculated in a commercially and validated software (ICM+, Cambridge Enterprise, University of Cambridge, UK) extended with a nICP software plugin, as previously reported in detail [21]. In short: the intracranial compartment is considered a black-box system, with ICP being a system response to the incoming signal ABP. This mathematical model provides a method to describe the transmission characteristics, with input and output signals. The intracranial compartment is modelized by a so-called impulse response function which connects the assumed input signal, ABP, with the output signal, ICP. Then, two linear models are established to depict the relationship between ABP and ICP (ABP→ICP model) and the relationship between ABP and FV with the application of certain TCD characteristics such as peak systolic, enddiastolic flow velocity and steepness of flow velocity increase and decrease, see for more detail [10]. The TCD characteristics may be derived from ABP and CBFV signals and, therefore, can be assessed noninvasively from the patient. The essential part of our nICP procedure is a description of the relationship between the TCD characteristics and the.

ABP → ICP model. A signal database including invasively assessed ICP of reference patients was used for this purpose. Therefore, the ABP → ICP model can be calculated from TCD characteristics, and its output data provides a continuous nICP waveform.

### Empty sella sign assessment

Brain MRI was performed using T1- weighted sagittal sequences to assess pituitary gland shape according to the classification proposed by Yuh et al. [8]. A concavity of the gland of more than one-third of the height of the sella was considered indicative of intracranial hypertension. This corresponds to categories III, IV, and V of the Yuh classification system and includes both empty and partially empty sella.

### Optic nerve sheath diameter (ONSD) determination

ONSD was assessed with B-mode using a Philips iU22 ultrasound system and a 9 − 3 MHz linear array transducer (Philips Medical Systems; Bothell, WA, USA). Examinations were done in a supine position with the upper part of the body and the head elevated to 20–30°. The mechanical index (MI) was reduced to 0.2, the thermal index to 0.0. The ultrasound probe was placed on the closed upper eyelid using ultrasound gel. The anterior part of the optic nerve was searched in a transversal plane showing the papilla and the optic nerve in its longitudinal course. ONSD was assessed 3 mm behind the papilla, as described previously [24]. ONSD was obtained as maximal diameter of the outer limits of the optical nerve sheaths and was obtained for the right and left side. Due to reference values of our neurophysiologic laboratory, values above 5.8 mm were assumed as pathologic [25].

### Lumbar puncture pressure assessment

Lumbar pressure measurement procedure followed standardized recommendations given in a consent statement paper [26]: Puncture was performed with an atraumatic 22 gauge lumbar puncture needle. Patients were positioned in a comfortable lateral decubitus position, with the vertebrae in line in the horizontal plane and the head in a neutral position on a pillow with the knees flexed. The needle was inserted in the midline of the spine, which is at the same level as the patient´s head. Aseptic technique is required as described in the articles. Lumbar punction was performed between the 4th and 5th spinous process. Once the needle is in the intradural space the stylet was withdrawn slowly waiting some seconds to see if liquor emerges. Once liquor is seen, the manometer (a three-way tap attached to the end of a commercially manometer (Pajunk, spinal manometer, Geisingen, Germany) is connected. After one minute the pressure is obtained, when the meniscus of liquor on top of the manometer oscillates with respiration. If liquor drainage is intended liquor is withdrawn by rotating the three-way-tap and collecting liquor in specimen bottles.

### Statistics

Sample calculation for correlation analysis was done with the G*Power 3.1 software with assumption of a large effect size, power of > 0.8, significance niveau *p* < 0.05 a number of 43 patients was calculated sufficient [27]. The effect size was calculated with G*Power from the coefficient of determination from a previous validation study [21].

For evaluations we used statistical software (StatView, Version 5.0.1., SAS Institute, North Caroline, USA). Based on the measurement level data were evaluated by paired and non-paired t-test and χ^2^ test, correlations were calculated according to the Pearson or Kendal test. Test results with probability *p* < 0.05 were considered significant.

Comparison between ONSD and LPP as well as nICP and LPP: Pearson correlation (R) was applied to pairs of corresponding ONSD or nICP and LPP values, respectively. Normal distribution of the differences was evaluated by Shapiro-Wilk test [17]. Outliers were assessed in terms of mean difference (MD) and their standard deviation (SD). The limits of agreement (LA) of probability *P* = 0.95 may be estimated with good accuracy by the interval (MD-2*SD, MD + 2*SD) in case of normal distribution. Deviations between non-invasive data and LPP were assessed in terms of mean difference and standard deviation, if applicable. The capability of nICP to assess LPP was assessed using ROC analysis of all data.

Comparison between Empty sella sign and LPP: LPP data were transformed in ordinal data as given: “no increased LPP” =0 or “increased LPP” =1 according to values lower or higher than 20 cm/H_2_O, Data were evaluated with non-parametric Kendall correlation.

In case of significant correlation between two data pairs the Bland-Altmann plot was calculated with upper and lower limits [28]. Besides the ROC analysis sensitivity, specificity and negative predictive values were calculated.

## Results

60 patients (sex ratio: 41 (68%) females, 19 (32%) males; age: 40 ± 13 years; BMI: 32 ± 9 kg/m^2^) had a complete set of data with evaluation for an empty sella, ONSD, nICP and LPP and were used for further evaluation. LPP was 21 ± 6 cmH_2_O. 12 patients (10 females; age: 43 ± 14 years; BMI 34 ± 9 kg/m^2^) had an increased LPP. No patients had to be excluded because of other reasons than IIH in the MRI scan or because of vascular disease or cardiac arrhythmia.

### Primary study (nICP-LPP) results

**Fig. 1:**
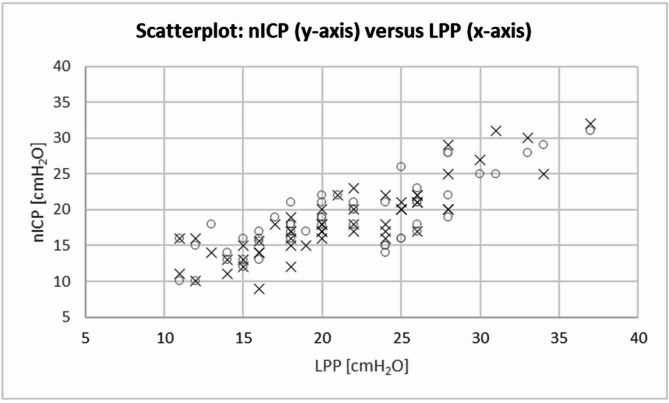
nICP vs. LPP. Data are given for the left (open circles) and right (crosses) sides. Data correlated significantly with *R* = 0.82 (*p* < 0.001) on the right, and *R* = 0.79 (*p* < 0.001) on the left side. nICP, non-invasive intracranial pressure; LPP, lumbar puncture pressure

**Fig. 2:**
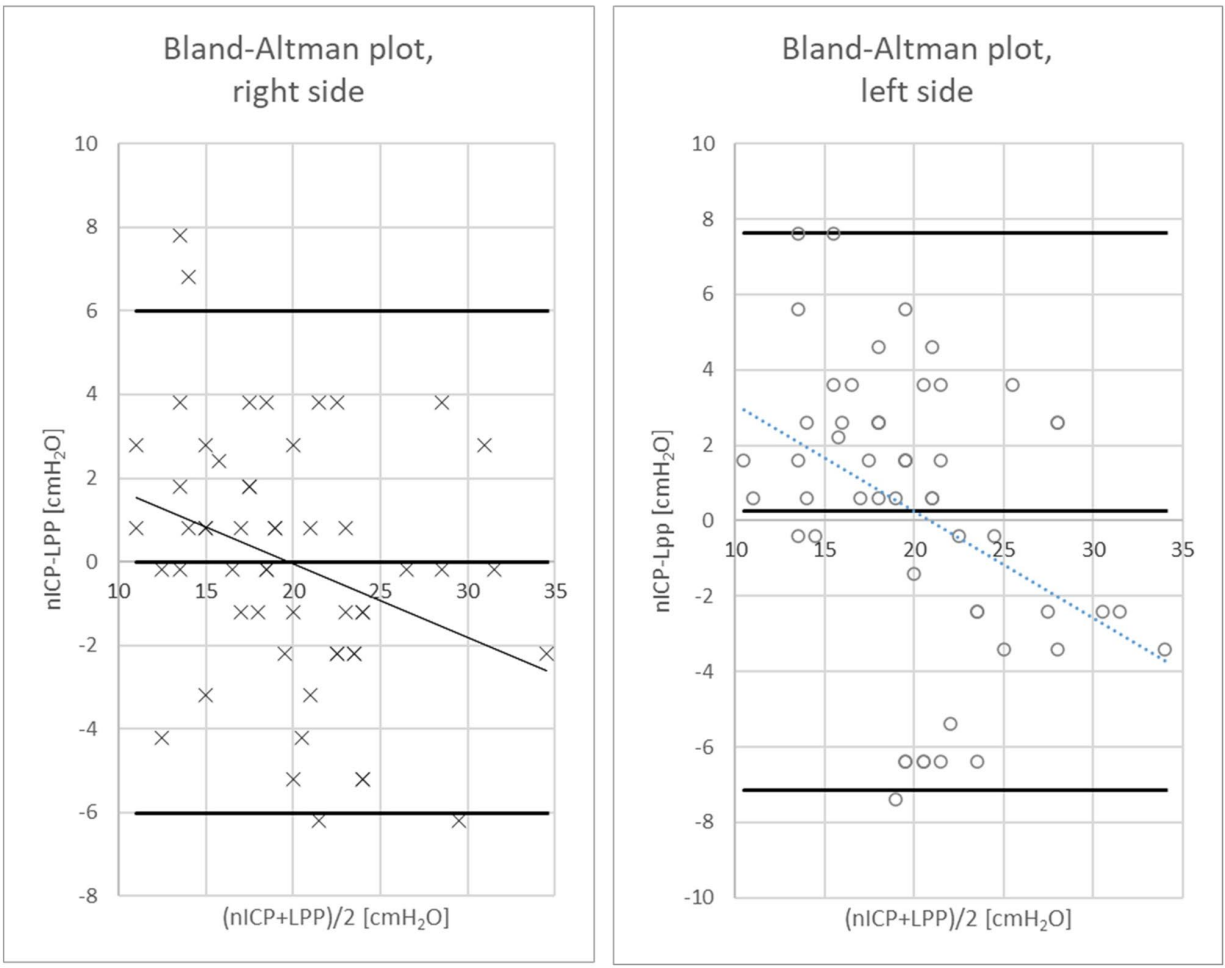
Bland-Altman Plot comparison between LPP and nICP. Corrected for the absolute pressure differences between methods, the MD ± SD of nICP-LPP for the right side (crosses) is -0.22 ± 3.1 cmH_2_O, for the left side (circles) is 0.24 ± 3.8 cmH_2_O. The difference LPP-nICP slightly increases with increasing pressure (nICP + LPP)/2 on both sides. On both sides the plot trend line intersects the line of equal LPP and nICP (nICP-LPP = 0) at a pressure close to 20 cmH_2_O. The Limits of Agreement are (-6.03, 5.99) for *P* = 0.95 on the right, and (-7.14, 7,61) for *P* = 0.95 on the left side. nICP, non-invasive intracranial pressure; LPP, lumbar puncture pressure

**Fig. 3:**
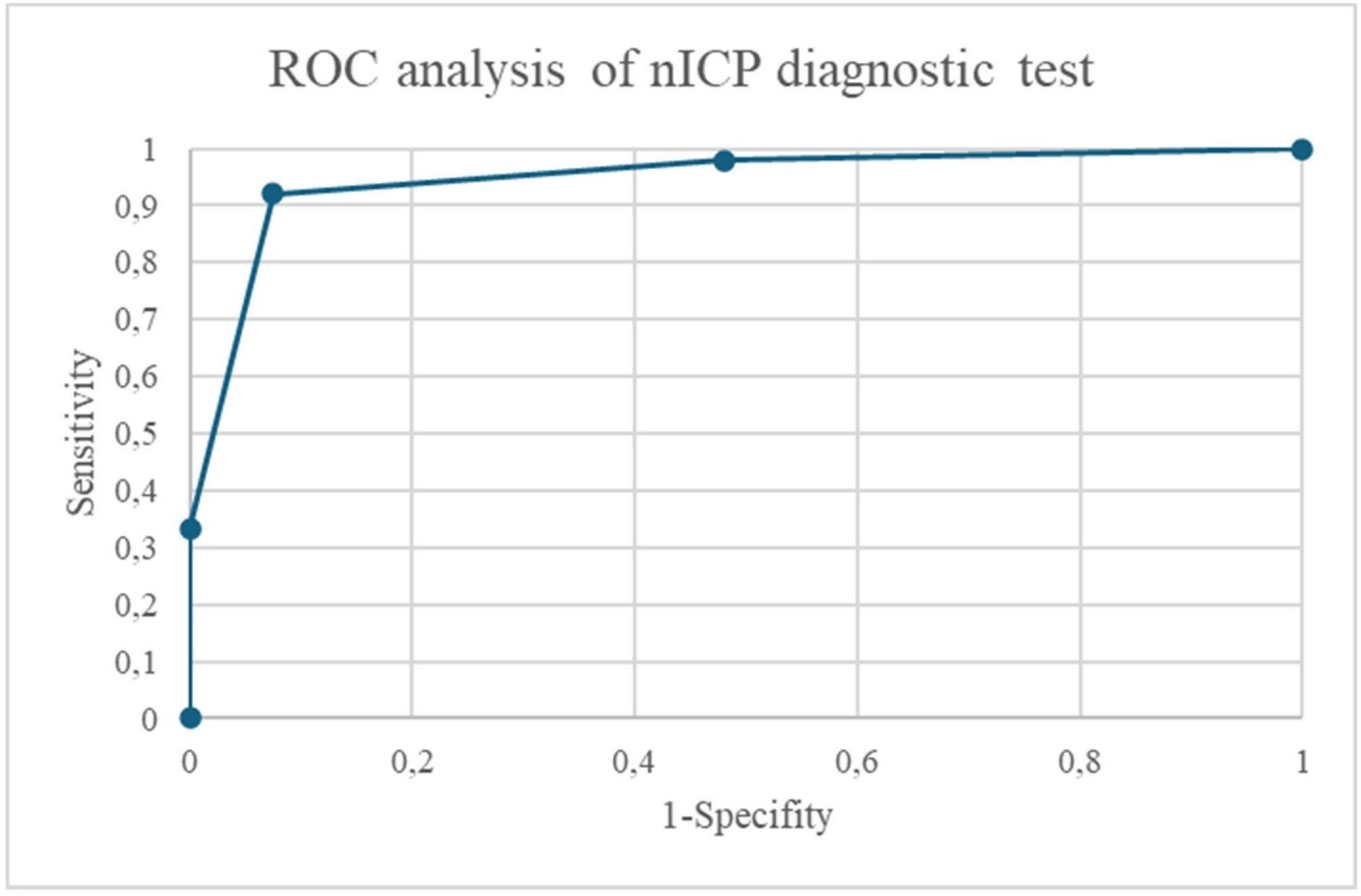
ROC Analysis for n-ICP diagnostic test. The arrow indicates the optimal cutoff for the prediction of increased LPP. The cutoff is nICP > 20 cmH2O with the sensitivity 0.92 and specifity 0.88. The area under the ROC curve is 0.92

nICP was 18 ± 5 cmH_2_O on the right side and 19 ± 5 on the left side. nICP values of both sides were correlated with each other with *R* = 0.85 [95%CI: 0.77,0.91] (*p* < 0.001). nICP correlated with the LPP on the right side with *R* = 0.82 [95%CI: 0.78,0.9] (*p* < 0.001) and on the left side with *R* = 0.79 [95%CI: 0.66,0.87] (*p* < 0.001), as shown in Fig. 1. Mean difference between nICP and LPP (nICP-LPP) was − 2.8 ± 3.1 cmH_2_O on the right side and − 2.6 ± 3.8 cmH_2_O for the left side. The regression line in the Bland-Altman plot showed a negative trend for higher ICP values (Fig. 2). The ROC curve is shown in Fig. 3, the area under the curve was 0.92. Using 20 cmH_2_O as the critical threshold for indication of increased intracranial pressure in the nICP method, its sensitivity was 0.92, specificity 0.88 and negative predictive value was 0.88.

### Exploratory (ONSD-LPP and empty sella sign-LPP) results

**Fig. 4:**
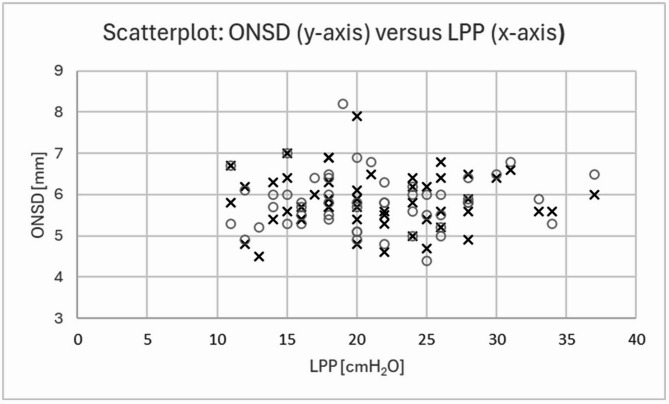
ONSD vs. LPP. Data are given for the left (open circles) and right (crosses) sides. No correlation was found between data. ONSD, optic nerve sheath diameter; LPP, lumbar puncture pressure

ONSD diameter was 5.9 ± 0.68 mm on the right side and 5.8 ± 0.67 mm on the left side. ONSD values of both sides correlated strongly with each other with *R* = 0.8 (*p* < 0.001). However, there was no correlation between LPP and the ONSD on the right (*R* = 0.04; *p* = 0.8) or left (*R* = 0.02; *p* = 0.9) side (Fig. 4). ONSD in patients with an LPP lower than 20 cmH2O was 5.9 ± 0.52 mm and in patients with values above 20 cmH2O was 5.8 ± 0.38 mm; there was no statistically significant difference between groups (*p* = 0.94).

The empty sella sign did not show a significant correlation to ordinal transformed LPP data (*p* = 0.48, n.s.).

## Discussion

From the presently investigated non-invasive ICP techniques, the nICP method had the highest agreement with the invasively determined LPP data. The correlation was about *R* = 0.8 (*p* < 0.001). The ROC curve showed a high AUC value of 0.82. As already demonstrated previously, we found a negative regression line of nICP and LPP data with higher values in the Bland-Altman plot pointing to a conservative assessment of the nICP method in the higher LPP range [21]. However, using a threshold of 20 cmH_2_O, we found a high sensitivity (0.92), specificity (0.91) as well as negative predictive value (0.93) of the nICP method. Therefore, we assume that the accuracy of the method is high enough to reduce the need for LP in patients with nICP values lower than 20 cmH_2_O. Technically, the nICP method seems to be clinically feasible since only a good flow velocity signal of the MCA is needed. The determination of the ABP with finger cuffs should then be no problem.

In line with our previous study, we found a highly significant correlation of nICP to LPP values [21]. Compared to the previous study, accuracy between LPP and nICP measurements could be improved from ± 4 cmH_2_O to ± 3 cmH_2_O [21]. This might be explained by the different techniques used to assess the blood pressure. In the former study a tonometric technique (Colin CBM 7000, ScanMed Medical Instruments, Moreton-in-Marsh, UK) was used in which a pressure sensor was placed on the radial artery. Presently, we chose a photoplethysmographic technique which obtains blood pressure data via body-size adjusted finger cuffs (Finapres nova, Enschede, Netherlands). The latter technique is less artefact sensitive, more comfortable for the patient and less dependent from obesity related tissue changes between the pressure sensor and the radial artery [29].

The mathematical evaluation in our study was done with a so-called black-box approach, although several other evaluation methods were used alternatively [30]. We used the technique for many years because of its repeatedly high prediction ability to detect increased ICP. In a recent study on the same field of investigation (patients with suspected idiopathic intracranial hypertension) we found a sensitivity and specificity in a ROC analysis of 0.92 [20, 21].

Although not primarily in the focus of our study, the lacking correlation of the other non-invasive parameters with the LPP should be addressed. The empty sella sign is found in up to 20% of routine MRI [31] and therefore seems not to be very specific for IIH. Consequently, our finding of a lacking correlation with the LPP data is line with previous MRI studies [32, 33]. Therefore, the empty sella sign should not be used as an indicator of an IIH.

Lacking correlation between ONSD and LPP data was in first sight surprising and cannot be related to technical artefacts since we have long standing experience with the method [24, 25]. Although the current consensus state recommends ONSD measurement without the dura mater [34], we do not think that this matter might have influenced the results. We explain the lacking correlation with the normal to moderately increased pressure levels in our suspected IIH patients. Similarly, a study in 139 patients with a similar ICP of 15mmHg (95% lower/upper confidence limit of 13/18 mmHg), i.e. 20.4 cmH_2_O (95% lower/upper confidence limit of 17.7/24.5 cmH_2_O), also failed to find a correlation of ONSD and LPP in men and reported only a weak correlation in women [35]. A recent population-based study with 579 patients also showed a weak correlation (*R* = 0.18, *p* = 0.01) between ONSD and ICP as assessed by LP (median LPP was 15.2 cmH_2_O with a range of 6 to 31.4 cmH_2_O) [36]. Therefore, pressure levels were assumed to be too low to result in a robust widening of the ONSD [36]. Good correlations between LPP and ONSD were mostly found in patients with confirmed and more severe IIH [37]: The authors studied patients with a LPP of 36.7 ± 11.8 cmH_2_O, which was much higher than that seen in our present work. Further research is warranted to investigate these mechanisms in more detail.

Limitations of the study might be that the LPP was taken as gold standard for obtaining intracranial pressure. Therefore, potential errors due to stress or tension induced false positive values could not be evaluated in this study. Also, false negative results due to an asymptomatic spinal canal stenosis or wall contact of the needle could have been missed. Many studies on IIH have been undertaken in pediatric patients. Although our approach should also be applicable in children this was not validated to the best of our knowledge, making further research necessary.

The highest accuracy to predict the LPP is around 20 cmH_2_O. In the range of pathologically increased or very low LPP levels the nICP is conservative showing less increased or less low ICP values. This is the reason for the wide upper and lower limits of agreement in the Bland-Altman Plots. The conservative calculation may be clinically irrelevant since nICP data will not normalize. However, the highest differences between nICP and LPP data around the 20 cmH_2_O threshold were approximately 7 cmH_2_O, which might be of clinical concern. However, we cannot determine in the current study whether the LPP or nICP data have caused the difference between values. To address this issue in more detail long term investigations in patients have to be determined which of the parameter might have a higher spread.

## Conclusions

Taken together, the TCD-based assessment of nICP seems to be a promising method for non-invasive diagnosis of ICP. Using a nICP threshold of 20 cmH2O the technique has a high accuracy to predict an increased ICP and might help in decision making to perform an invasive lumbar puncture. Due to its non-invasive nature, the nICP method might allow patient-friendly long-term monitoring of ICP. This is an important issue in patients with idiopathic intracranial hypertension since it is a chronic disease with long term treatment with medication or repeated lumbar drainage of liquor. It appears that in normal or moderate increased LPP conditions, the nICP might be more sensitive than the ONSD technique. Further investigations have to follow to further determine the conclusions of the present investigation.

## Data Availability

Owing to local privacy policy conditions data are not publicly available. In case of interest, a request should be sent to the corresponding author.
